# Genome Analysis Reveals a Synergistic Mechanism of Ursodeoxycholic Acid and Jasminoidin in Mice Brain Repair After Ischemia/Reperfusion: Crosstalk Among Muti-Pathways

**DOI:** 10.3389/fphar.2019.01383

**Published:** 2019-12-12

**Authors:** Yingying Zhang, Haixia Li, Huan Guo, Bing Li, Zide Zhao, Pengqian Wang, Hongli Wu, Jun Liu, Yinying Chen, Xiaoxu Zhang, Ping Wu, Zhong Wang, Jie Wang

**Affiliations:** ^1 ^Dongzhimen Hospital, Beijing University of Chinese Medicine, Beijing, China; ^2^Institute of Basic Research in Clinical Medicine, China Academy of Chinese Medical Sciences, Beijing, China; ^3 ^Guang’anmen Hospital, China Academy of Chinese Medical Sciences, Beijing, China; ^4^Eye Hospital, China Academy of Chinese Medical Sciences, Beijing, China; ^5^Institute of Chinese Materia Medica, China Academy of Chinese Medical Sciences, Beijing, China

**Keywords:** cerebral ischemia, synergistic mechanism, GeneGo MetaCore™ software, signaling pathway, network analysis

## Abstract

Studies have shown that combination drug therapy which corresponding treatment involves multiple genes and targets is more effective against cerebral ischemia. To identify the synergistic mechanism of ursodeoxycholic acid and jasminoidin based on differential pathway network, which protect against brain ischemia-reperfusion injury. Totally 115 mice with focal cerebral ischemia-reperfusion injury were allocated into five groups: sham, vehicle, ursodeoxycholic acid (UA), jasminoidin (JA), and JA and UA combination group (JU). The differentially expressed genes identified by microarray which consisted of 11,644 complementary DNAs were loaded to the GeneGo MetaCore™ software to analyze the enriched pathways and processes among different groups. Of the top 10 pathways and process networks, 5, 6, and 3 overlapping pathways as well as 5, 3, and 4 overlapping process networks were observed between UA and JA, UA and JU, and JA and JU, respectively. Of these, three pathways and three process networks overlapped across the three groups. Interestingly, four representative pathways and six process networks were only noted in the JU group. Gene Ontology process analysis showed 2 processes were shared by all three treatment groups in the top 10 processes. The UA and JA combination resulted in synergistic effects through affecting multi-signal transduction pathways, different locations in the same pathway, and the new signaling pathway emerged in drug combination group, those together may enhance the treatment of cerebral ischemia-reperfusion injury through promoting neural cell apoptosis, decreasing calcium levels, inhibiting inflammation, and protecting neurons.

## Introduction

Cerebral ischemia is one of the leading lethal disorders worldwide (Go et al., 2014). Its pathological process involves a series of biochemical and molecular changes (Mehta et al., 2007). Studies have shown that combination drug therapy is more effective against cerebral ischemia (Els et al., 2006; Smadja et al., 2011; O’Collins et al., 2012).

The Chinese medicine Qingkailing injection, which includes baicalin (BA), ursodeoxycholic acid (UA), jasminoidin (JA), and *Concha margaritifera* (CM) has been shown to display protective effects against focal cerebral ischemia and reperfusion injury (Zhang et al., 2006). It has diverse components with pharmacodynamic characteristics (Zhou et al., 2010), of which synergistic effect noted with the JU (JA-UA combination) treatment, such as decreased infarction volumes, recovery of the neurological deficit score and MRI findings in ischemic mice (Koo et al., 2006; Zhang et al., 2006; Zhou et al., 2010). Our previous study has revealed a partial pathway- and network-based transformation of the synergistic mechanism of JA and UA against cerebral ischemia-reperfusion injury by using the Kyoto Encyclopedia of Genes and Genomes pathways analysis and Ingenuity Pathway Analysis (IPA). This study aimed to further investigate the mechanisms by which UA and JA combination protects against brain ischemia with GeneGo MetaCore^™^ software.

GeneGo MetaCore^™^ is a pathway analysis tool based on a proprietary manually curated database of various high throughput data (Baitaluk et al., 2006). It elucidates and visualizes the underlying molecular mechanisms and pathways of complex disorders (Ekins et al., 2007). Here, GeneGo MetaCore^™^ was used to further analyze the mechanism of the UA-JA combination in protecting against cerebral ischemia-reperfusion injury.

## Materials and Methods

### Animal Model

One hundred and fifteen specific pathogen-free healthy adult male Kunming mice (12-week-old, 38–48 g) were placed at a constant temperature of 25°C and were subjected to a 12-12 h light-dark cycle. All experiments complied with the Prevention of Cruelty to Animals Act (1986) and National Institutes of Health (NIH) guidelines for the care and use of laboratory animals. The experimental protocol gained approval from a local committee review.

After anesthesia with 2% pentobarbital (4 mg/kg, i.p), surgical operation to induce middle cerebral artery occlusion (MCAO) was performed. Focal cerebral ischemia-reperfusion was induced by occluding the left middle cerebral artery for 90 minutes with an intraluminal filament followed by 24 h of reperfusion. Sham animals were treated in the same manner except that the filament was not inserted. Throughout the surgical procedure, the rectal temperature was kept at 37.0–37.5°C by use of a heating pad; brain temperature was monitored with a thermocouple implanted in the right striatum and was maintained at 36–37°C using a thermostatically controlled lamp. Besides, blood pressure, blood gas, glucose levels, and EEGs were also monitored during operation. Infarction volume and subsequent mice behavior for each mouse was used to assess the operation success according to the method described by Bederson et al. (Bederson et al., 1986a; Bederson et al., 1986b; Wang et al., 2011).

### Animal Grouping and Treatment

The experimental animals were divided into five groups (n = 23): sham, vehicle (0.9% NaCl), UA (ursodeoxycholic acid; 5 mg/ml), JA (jasminoidin; 25 mg/ml), and JU (combination of ursodeoxycholic acid and JA at a ratio of 1:1) treatment groups. The herbal preparation was dissolved in 0.9% NaCl immediately before use. All preparations were injected intravenously *via* the tail vein at a dose of 2 ml/kg body weight.

### Ribonucleic Acid Extraction and Microarray Experiments

The left hippocampi of five mice from each group were homogenized in TRIzol^®^ Reagent (Invitrogen, CA, USA). Total RNA was extracted using the RNeasy Micro Kit (Qiagen, Valencia, CA, USA) following the protocol supplied by the manufacturer. RNA quality was evaluated by analyzing the 26S/18S ratio with Bioanalyzer microchip (Agilent, Palo Alto, CA, USA). Microarrays were created from a total of 374 cerebral ischemia-related complementary DNAs. The collection procedure of 11,644 genes, as well as the detailed protocols for RNA extraction and microarray sample preparation have been described in our prior study (Chen et al., 2012).

### Microarray Analysis

All experimental data were uploaded to the ArrayTrack system (FDA, USA). All microarray data obtained were normalized with locally weighted linear regression (Lowess) to reduce the variability in datasets (Koo et al., 2004). In order to make pairwise comparison of the means of the altered genes between vehicle and sham, UA and vehicle, JA and vehicle, and JU and vehicle groups, one-way analysis of variance (ANOVA), and significant analysis of microarrays were performed. Genes with a p value < 0.05 and a fold change >1.5 were screened out, and then a Bonferroni correction was conducted to select a list of significant genes for further analysis. A >1.5-fold increase or <0.5-fold decrease in the expression levels indicated up- or down-regulation, respectively. The details have been described in our prior studies (Wang et al., 2011; Li et al., 2013; Li et al., 2016). Afterwards, all significantly differentially expressed genes were uploaded to the software packages MetaCore^™^ (GeneGo, St. Joseph, MO, USA, Version: 2012, 07). Enrichment analysis is a useful tool that determines the functional distribution and the significantly enriched functional categories (p-value <0.1) of the genomic/proteomic expression profile. It would help to understand the functions of these differentially expressed genes and offer essential information for further bioinformatics analysis. The biological process, sub-cellular location, and functional distribution of the differentially expressed genes were computed using MetaCore^™^ based on Gene Ontology. The network construction and analysis were done by use of the analyze network algorithm provided in the software. And network distribution of the selected genes was also determined *via* MetaCore based on GeneGo network ontologies. The enrichment p-values in result tables were calculated based on EASE Score, which is a rigorously modified Fisher exact test p value. Statistical relevance of the identified ontology matches was calculated as p-value, or a probability of a match to occur by chance. The more genes/proteins belong to a process/pathway, the lower the p-value.

### Western Blotting

The expression levels of JunB proto-oncogene (JUNB) were measured using Western blotting. The hippocampus tissue of 15 mice from five groups was excised from the brain at 24 h post-ischemia. Following cell lysis, 40 mg of protein were subjected to electrophoresis on 10% sodium dodecyl sulfate-polyacrylamide gels and then transferred to polyvinylidene difluoride membranes. Next, the primary and secondary antibodies rabbit anti-JUNB (CST3753) and mouse anti-b-actin (Tiandeyue, Beijing, China) were applied. Optical density-calibrated images were captured with a video camera for analysis. All measurements were performed in triplicate. The JUNB protein expression levels were calculated by normalization to b-actin levels.

## Results

### Pharmacodynamic Results

It has been shown that the JA-UA combination had a pharmacodynamic synergistic effect in treating cerebral ischemia (Koo et al., 2006; Guo et al., 2014).

### Pathways Enriched in Three Treatment Groups

**Figure 1:**
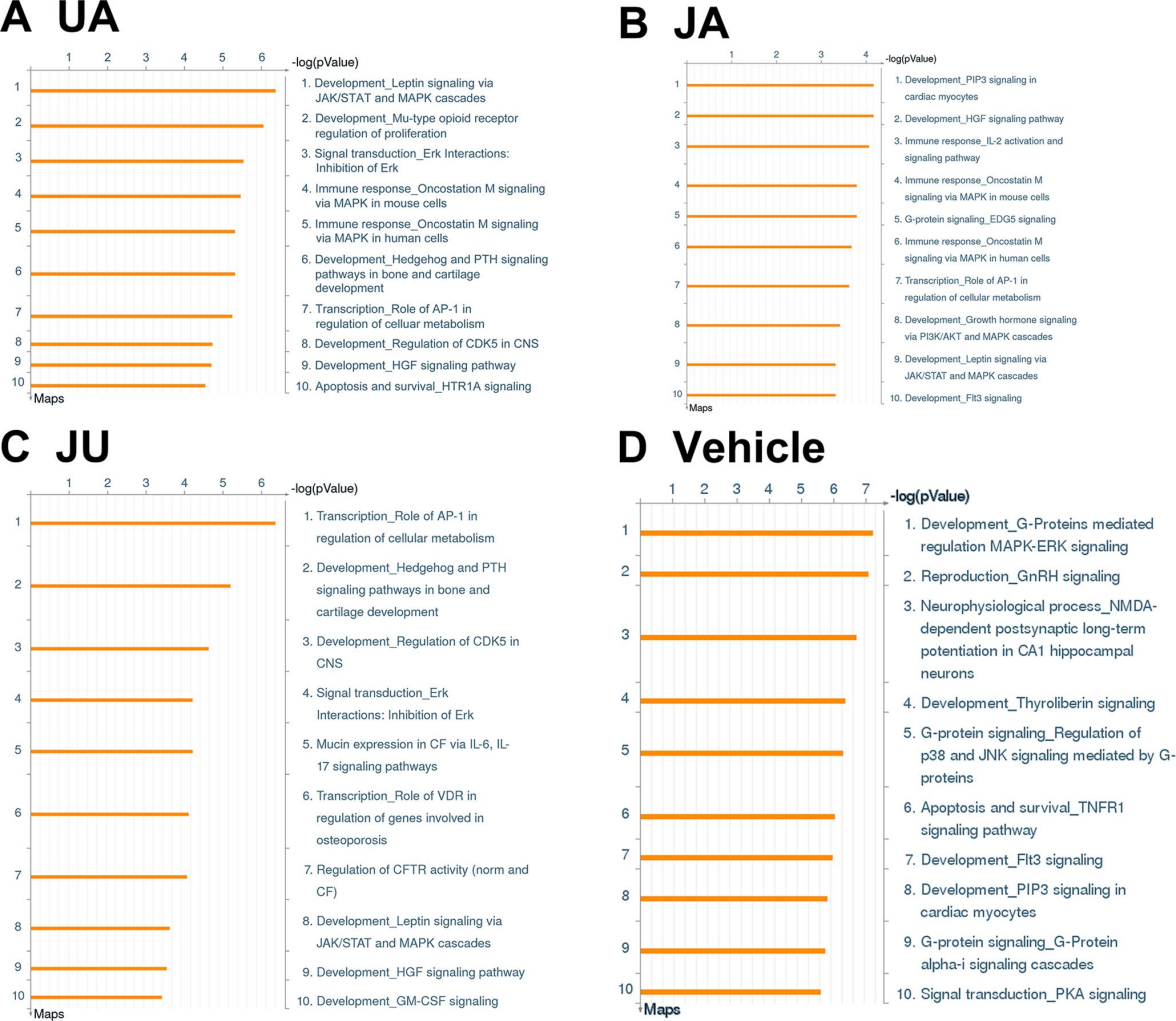
Pathways involved differential expressed genes after ursodeoxycholic acid (UA), jasminoidin (JA), and JA and UA combination group (JU) treatments compared with model group. **(A)** UA group. **(B)** JA group. **(C)** JU group. **(D)** Vehicle group.

Based on microarray data analysis, we found 414, 470, and 401 differential expression genes in JA, UA, and JU group, respectively. Compared to sham group, the enrichment pathways in vehicle and treatment groups were mostly focus on functions such as development, signal transduction, immune response, transcription, G-protein signaling, and apoptosis and survival, and the top 10 signaling pathways in each group were shown in **Figure 1**.

**Figure 2:**
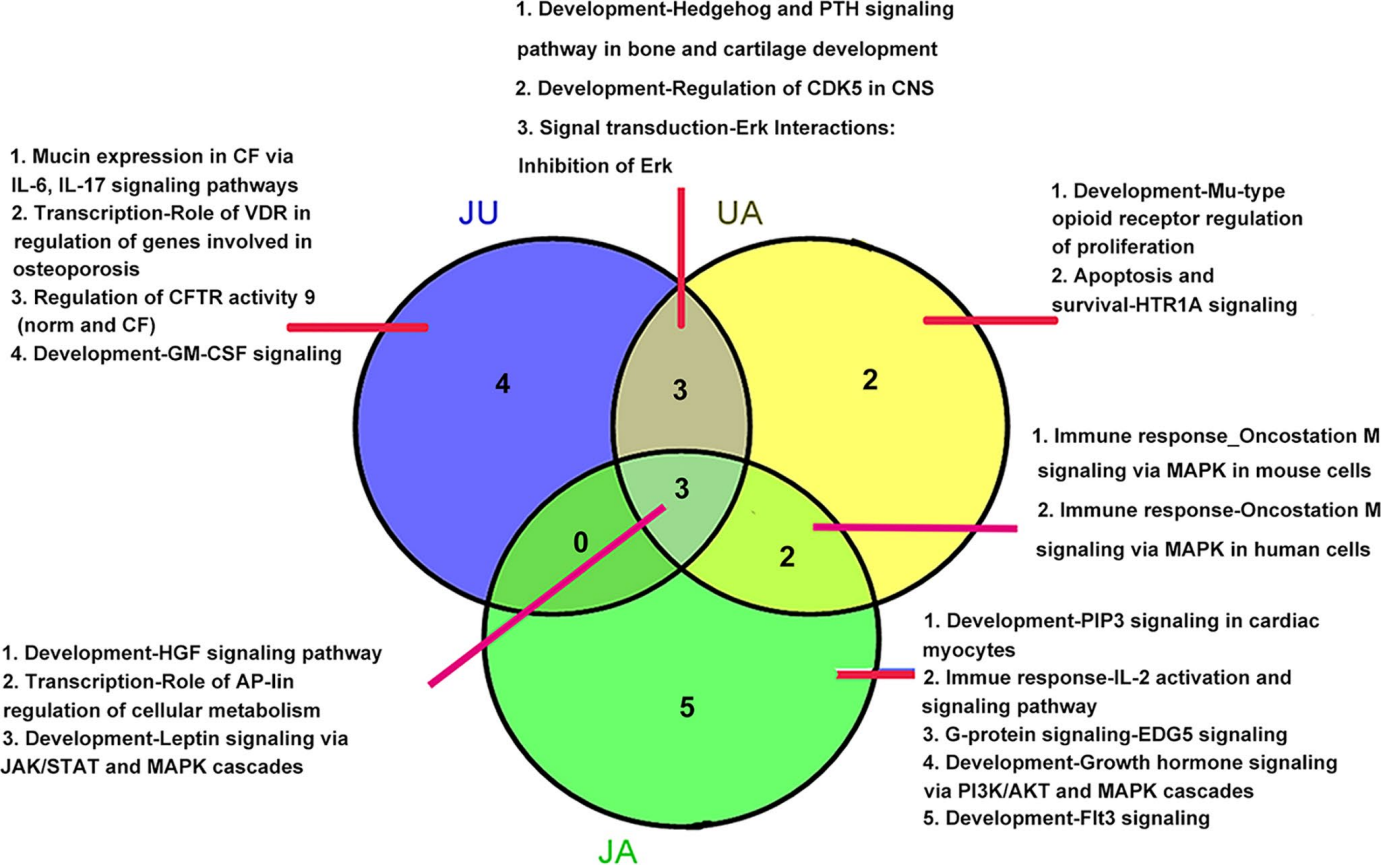
Overlapping pathways among different group.

There were three overlapping pathways in three treatment groups, including development-hepatocyte growth factor signaling pathway, development-leptin signaling *via* Janus kinase/signal transducers and activators of transcription (JAK/STAT) and mitogen-activated protein kinase (MAPK) cascades, and transcription-role of activator protein 1 (AP-1) in regulation of cellular metabolism. In addition, three overlapping pathways were found between UA and JU groups. Meanwhile, there were specific pathways in each treatment group, for example, there were four specific pathways in JU groups, including Mucin expression in CF *via* IL-6, IL-17 signaling pathways, transcription-role of vitamin D receptor (VDR) in regulation of genes involved in osteoporosis, regulation of cystic fibrosis transmembrane conductance regulator (CFTR) activity 9 (norm and CF), development-granulocyte-macrophage colony-stimulating factor (GM-CSF) signaling (**Figure 2**).

### Gene Modulation in Common Pathways Altered in Ursodeoxycholic Acid and Jasminoidin and Ursodeoxycholic Acid Combination Group

**Figure 3:**
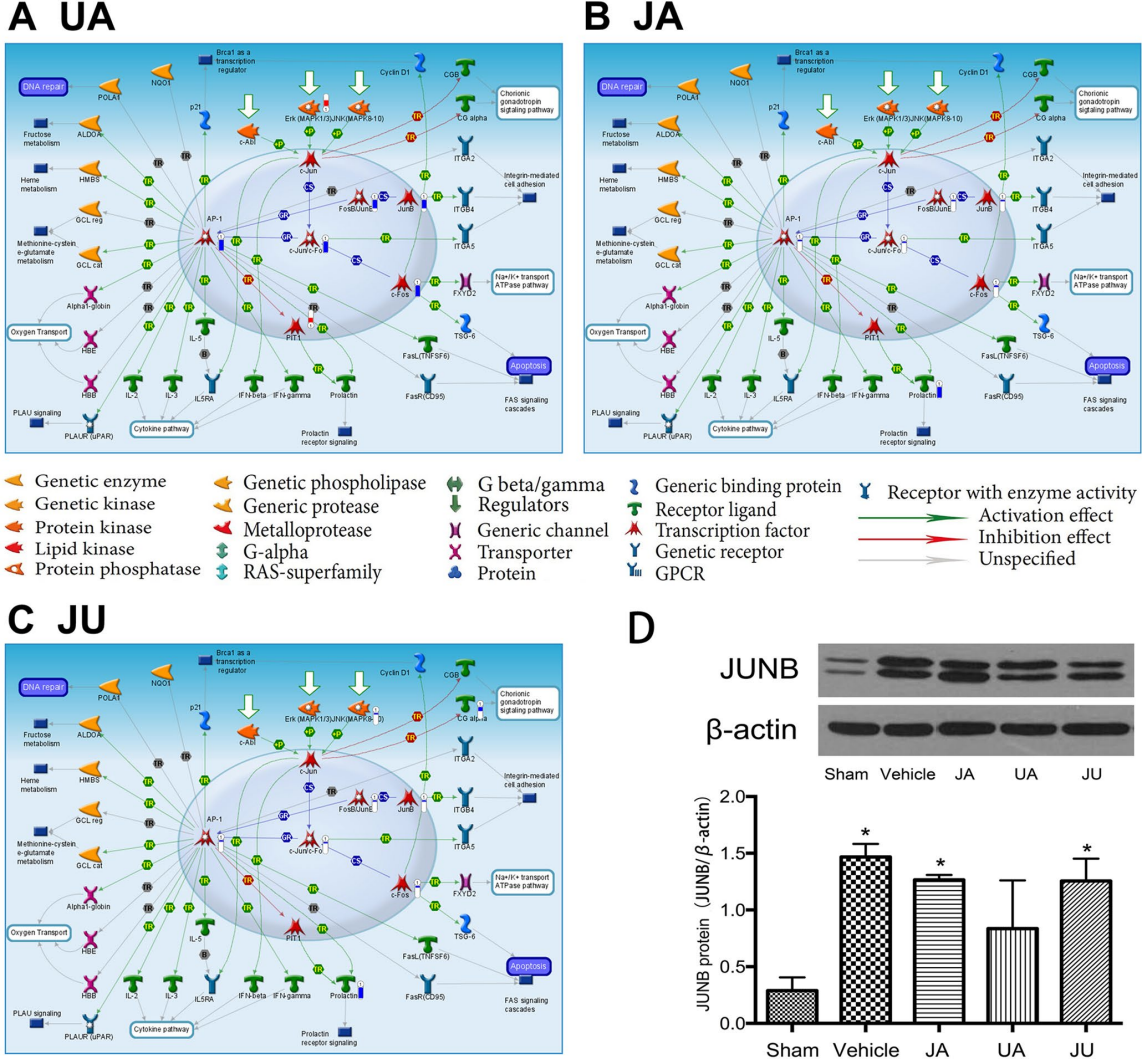
Differentially expressed genes in eTranscription_Role of activator protein 1 in regulation of cellular metabolism” of different treatment groups. **(A)** Ursodeoxycholic acid group. **(B)** Jasminoidin group. **(C)** JA and UA combination group. **(D)** Biological verification of JunB proto-oncogene by Western blot, *P < 0.05 vs. sham.

Though there were common pathways in three treatment group, the moleculars in pathway affect by different drug showing the difference. For example, the pathway of “Transcription_Role of AP-1 in regulation of cellular metabolism” was the most significant enriched pathway in JU, in which eight genes were significantly differentially expressed, the differential expression genes of the three groups in this pathway were showed in **Figure 3**.

### GeneGo Processes Enriched in Three Treatment Groups

**Figure 4:**
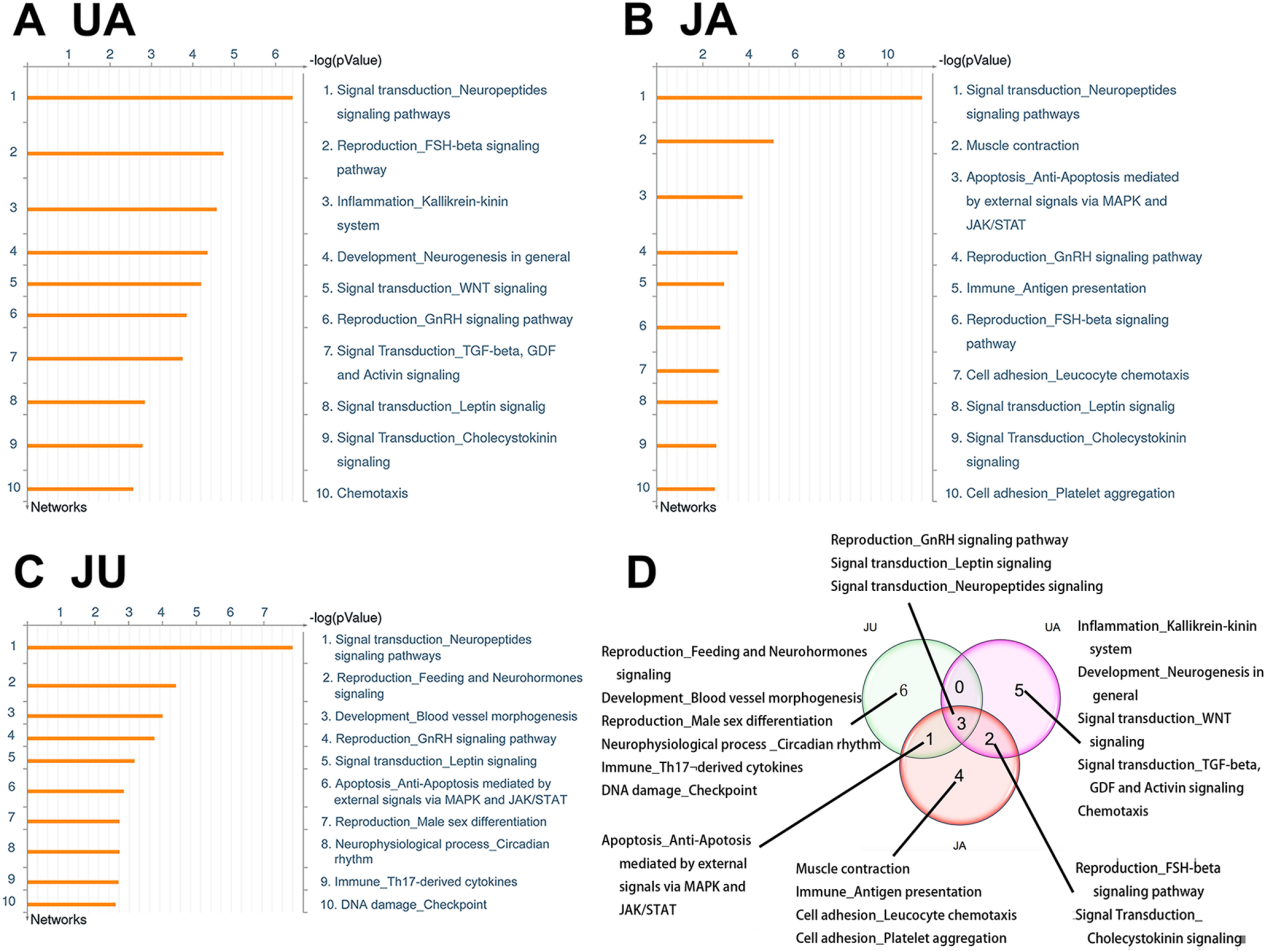
The top 10 GeneGo processes in 3 treatment groups.

Similarly, the top 10 GeneGo processes that were enriched by the differentially expressed genes in the UA, JA, and JU groups were listed in **Figure 4**, respectively. There are three common processes shared in three treatment groups, including Signal transduction_Leptin signaling, Reproduction_GnRH signaling pathway, and Signal transduction_Neuropeptides signaling pathways. And 1 common process shared in JU and JA is Apoptosis_Ant-Apoptosis mediated by external signals *via* MAPK and JAK/STAT.

### Variation of Targets in the Same Process After Different Treatments

**Figure 5:**
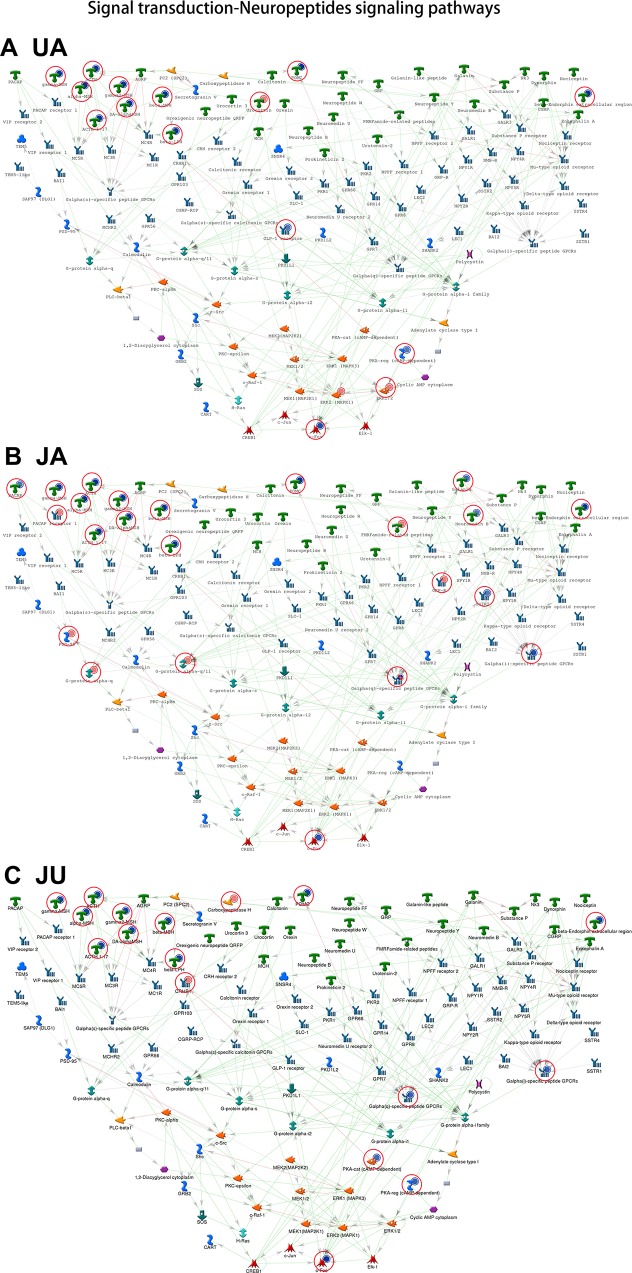
The common process of treatment groups. Signal transduction-neuropeptides signaling pathways. **(A)** Ursodeoxycholic acid group. **(B)** Jasminoidin group. **(C)** JA and UA combination group.

Of the top 10 ranked enriched processes in the three groups, a common process called “*signal transduction-neuropeptides signaling pathways*” consisted of 31 molecules, with 16, 22, and 17 altered in UA, JA, and JU groups, respectively (**Figure 5**). Of these molecules, gamma-melanocyte-stimulating hormone (MSH), adrenocorticotropic hormone (ACTH), alpha-MSH, gamma2-MSH, dopamine-alpha MSH, ACTH1–17, beta-MSH, beta-lipotropic hormone, pro-opiomelanocortin, and c-Fos were activated by all of three groups. In the JU group, the up-regulated molecules included G-alpha(q)-specific peptide G-protein-coupled receptors (GPCRs) and protein kinase A catalytic subunit (PKA-cat) (cAMP-dependent); carboxypeptidase H and CRHR1 were down-regulated. In JU and JA treatment groups, the up-regulated molecules included G-alpha(i)-specific peptide GPCRs. PKA-cat (cAMP-dependent) molecules were up-regulated in both JU and UA treatment groups.

### Sub-Network Analysis

No identical sub-network was obtained between UA and JA, UA and JU, and JA and JU comparison groups. JU combination appeared to significantly affect the skeletal system of sub-networks, including the five nodes FUCO: 1,2-propanediol oxidoreductase, DGCR8, CRIM1, and COL1A1 (**Figure 4**). Besides, combination of JA and UA starkly affected the regulation of adenylate cyclase and cAMP biosynthetic and metabolic processes (**Figure 5**, **Table 1**).

**Table 1:** Network and Gene Ontology process of differential expressed genes.

No	Network	GO Processes	Total nodes	Root nodes	Pathway	p-Value	z-Score	gg-Score
**1**	FAK, o-Src, PAK1, paxilin, fibronectin	Localization of cell (60.4%: 7.163e−29). Cell motion (60.4%: 7.163e−29), celluar component organization (87.5%: 2.142e−25). Cell projection organization (52.1%: 2.972e−25). Anatomical structure for motion (66.7%: 2.287e−23)	51	39	711	2.00E−35	19.56	908.31
**2**	Caspase-3, caspase-7, o-IAP1, caspase-6, caspase-9	Apoptosis (63.8%: 7.982e−31). Programmed cell death (63.8%: 1.303e−30). Cell death (63.8%: 5.837e−29). Death (63.8%: 7.940e−29). Induction of programmed cell death (46.8%: 1.135e−24)	50	29	704	6.17E−21	14.05	894.05
**3**	ESR1 (nuclear), ERK2 (MAPK1), SMAD3, TFF1, ERK1/2	Response to stimulus (75.0%: 1.944e−12). Response to chemical stimulus (54.2%: 9.653e−12). Response to steroid hormone stimulus (27.1%: 1.306e−11). Response to hormone stimulus (31.2%: 4.942e−11). Response to estrogen stimulus (20.8%: 5.308e−11)	50	36	329	3.54E−31	18.13	429.38
**4**	STAT3, STAT1, STAT5, o-Fos, JAK2	Response to chemical stimulus (74.0%: 5.601e−23). Response to molecule of bacterial origin (34.0%: 1.180e−22). Response to lipopolysaccharide (32.0%: 1.318e−21). Protein kinase cascade (44.0%: 6.273e−21). Positive regulation of cellular process (68.0%: 5.192e−19)	50	41	212	7.59E−39	20.67	285.67
**5**	o-Myc, SMAD3, o-Jun, p53, SMAD4	Regulation of developmental process (80.0%: 9.932e−30). Positive regulation of cellular process (78.0%: 2.831e−25). Multicellular organismal development (90.0%: 1073e−24). Organ development (78.0%: 1.719e−24). Positive regulation of developmental process (58.0%: 3.131e−24)	50	35	186	3.76E−29	17.36	249.86
**6**	o-Myc, AKT(PKB), IRS-1, p70s6 kinase1, IGF-1 receptor	Insulin receptor signaling pathway (33.3%: 3.495e−29). Transmembrane receptor protein tyrosine kinase signaling pathway (41.7%: 1.670e−22). Enzyme linked receptor protein signaling pathway (41.7%: 4.297e−19). Response to insulin stimulus (25.%: 5.338e−16). Phosphoinositide 3-kinase cascade (14.6%: 7.933e−16)	50	30	134	1.40E−20	14.79	182.29
**7**	CREB1, Oct-3/4, CBP, RARalpha, TGF-beta receptor type II	Response to stimulus (84.0%: 1.447e−17). Reproductive process (48.0%: 2.783e−16). Reproduction (48.0%: 3.303e−16). Transforming growth factor beta receptor signaling pathway (24.0%: 1.382e−15). Transmembrane receptor protein serine/threonine kinase signaling pathway (24.0%: 3954e−15)	50	42	53	1.22E−40	21.22	87.47
**8**	ESR1(nuclear), p21, STAT5B, caveolin-1, CDK2	Response to stimulus (90.0%: 2.383e−21). Response to stress (50.0%: 4.733e−10). Response to endogenous stimulus (30.0%: 5.046e−10). Response to hormone stimulus (28.0%: 1.122e−09). Response to chemical stimulus (46.0%: 9.735e−09)	50	45	29	2.05E−46	22.88	59.13
**9**	NK-kB, HIF1A, STAT1, o-Myc, p53	Response to stimulus(92.0%: 8.985e−23). Response to stress (70.0%: 4.893e−20). Immune system process (56.0%1.296e−18). Response to bacterium (36.0%: 1.383e−18). Response to biotic stimulus (42.0%: 2.111e−18)	50	46	25	1.68E−48	23.43	54.68
**10**	GCR-alpha, P27kip1, ETS1, NF-Kb, ERK1/2	Positive regulation of biological process (66.7%: 1.495e−12). Positive regulation of cellular process (63.9%: 2.048e−12). Regulation of cell proliferation (47.2%: 1.601e−11) Regulation of location (41.7%: 3.433e−11). Response to stimulus (77.8%: 1.327e−10)	50	28	31	1.37E−23	15.52	54.27

### Gene Ontology Process Analysis

In the top 10 Gene Ontology (GO) processes (**Table 1**), 5 were found in UA and JU groups; JA and JU groups shared 2 GO processes; 2 GO processes were shared by all three treatment groups. More overlapping processes were found between UA and JU group compared with those of the JA and JU group. The pharmacological mechanism was altered after drug combination, as indicated by the occurrence of new processes.

### Biological Verification of JunB Proto-Oncogene by Western Blot

The Western blot analysis indicated that, in comparison with the sham group, the expression level of JUNB was significantly up-regulated in the vehicle, JA and JU groups (P < 0.05) (**Figure 3D**). But compared with the vehicle group, the expression level of JUNB showed a trend of down-regulation in JA, UA, and JU groups, and the level of down-regulation in UA was more than that in JA and JU groups, consistent with the findings of Microarray data analysis and GeneGo MetaCore™ software (**Figures 3A–C**).

## Discussion

Our study offers a novel combination of microarray analysis with MetaCore, which may provide an in-depth analysis of the detailed and specific changes in gene expression and pathways associated with cerebral ischemia-reperfusion injury.

In the pathways shared by all three treatment groups, taking the transcription-role of AP-1 in regulation of cellular metabolism pathway as an example, the expression of AP-1, c-JunrVc-Fo, JunB, FosB-JunB, and c-Fos is regulated through extracellular-signal-regulated kinase (Erk) downregulation. Excitatory amino acids, calcium overload, free radicals, and increased NO all involve the expression of c-Fos in the central nervous system during brain ischemia/reperfusion. c-Fos can combine with c-Jun to form the AP-1 complex, which affects the transcription of genes involved in brain ischemia/reperfusion injury, thus promoting neural cell apoptosis (Liu et al., 2007). In our previous studies, the JA-UA combination demonstrated a synergistic effect in treating cerebral ischemia. Hundreds of differentially expressed genes were detected in each group using microarray experiments. Due to the overwhelming number, we have chosen some important molecules to verify using PCR or western blotting, such as IL-6, Creb, ERK1, ERK2, Guk1, Hrsp12, Mrm1, Atf2, Fos, FosB, Cebpg, Hspa1a, and Rara (Wang et al., 2011; Liu et al., 2012; Zhang et al., 2014; Chen et al., 2016). All of these were defined as differentially expressed genes by both microarray experiments and PCR or western blotting. This time the biological verification of JUNB by Western Blotting indicated that the level of down-regulation in UA group was greater than that of JA and JU groups, in line with the findings of microarray data analysis and GeneGo MetaCore^™^ software.

Another example is the development-Hedgehog and parathyroid hormone (PTH) signaling pathways in bone and cartilage development pathway, which is a common pathway between UA and JU (**Figure 1**). In JU group, this pathway not only regulates PTH components, but also elevates blood calcium, decreases calcium levels in cells, increases the expression of vascular endothelial growth factor, stromal cell-derived factor 1, and brain-derived neurotrophic factor, enhances the newly formed vessels in the peri-infarct cortex, promotes neuroblast migration and increases the number of neurons in cortex (Sato et al., 2003; Wang et al., 2014). Besides, it can also relieve inflammation by regulating the levels of VDR (Autier and Gandini, 2007) and indirectly improve the adverse effects of cerebral ischemia due to unfavorable physical activity that may cause osteoporosis (Cui et al., 2011).

Different from JA or UA monotherapy, in the top 10 pathways altered in JU treatment, 5 were related to development processes and 2 to transcription, and others were involved in signal transdection, Mucin expression in CF *via* interleukin 6 (IL-6), IL-17 signaling pathways, and regulation of CFTR activity. The development-GM-CSF signaling pathway was specifically altered by JU. GM-CSF plays an important role, inhibiting inflammation and protecting neurons (Cheng and Zhao, 2004; Rizk et al., 2006), indicating its potential usefulness in treating ischemic brain injury. We identified the GM-CSF alpha-receptor as an up-regulated gene in a screening for ischemia-induced genes in the cortex. This receptor is expressed widely throughout the brain, and can be induced by ischemic injuries. In primary cortical neurons and human neuroblastoma cells, GM-CSF acts against programmed cell death and induces BCL-2 and BCL-Xl expressions (Schabitz et al., 2008). Of the signaling pathways analyzed, GM-CSF most significantly induced the PI3K-Akt pathway, and inhibition of Akt significantly reduced anti-apoptotic activity. Intravenous administration of GM-CSF was shown to pass the blood-brain barrier, and decrease infarction volume in two different stroke models (Schabitz et al., 2008). GM-CSF may offer useful insights into the development of novel drug candidates for stroke and neurodegenerative diseases (Hossmann and Buschmann, 2005).

Beside signaling pathways, variation of targets in the same process occurred after different treatments; for example, “signal transduction-neuropeptides signaling pathway,” different target genes were affected according to the drug used. The up-regulated genes were very similar but those down-regulated differed considerably. Thus, the UA and JA combination not only changes the original processes but also uses different molecular mechanisms when acting on the same process.

## Conclusion

The UA and JA combination resulted in synergistic effects through affecting multi-signal transduction pathways and different locations in the same pathway, besides that, new signaling pathway also emerged in the drug combination group. All of these may adjust the mechanisms involved in treating cerebral ischemia-reperfusion injury in all aspects for improvement, including promoting neural cell apoptosis, decreases calcium levels, inhibiting inflammation, and protecting neurons, the multi-targets, and multi-channel effects of the combination should provide accelerated recovery in cerebral ischemia.

## Ethics Statement

The animal experiments were carried out in accordance with the Prevention of Cruelty to Animals Act (1986) and National Institutes of Health (NIH) guidelines for the care and use of laboratory animals for experimental procedures. The experimental protocol was approved by a local committee review.

## Author Contributions

ZW and JW designed the study. YZ and HL wrote the manuscript; HG, BL and XZ performed the research; YZ and ZZ modified tables and figures; PWa, HW, JL ,YC and PWu analyzed the experimental data. All authors participated in the discussion and data analysis and reviewed the manuscript.

## Funding

This study was partially funded by the Basic Research Fund of Guest Professor Innovation Research Project in China Academy of Chinese Medical Sciences (No. ZZ070803), and partially by the Natural Science Foundation of China (NSFC) (No. 81303132).

## Conflict of Interest

The authors declare that the research was conducted in the absence of any commercial or financial relationships that could be construed as a potential conflict of interest.
